# Supplementary material for: Climate’s influence on topography encoded in stream network topology and geometry
Source: Nat Commun. 2026 Mar 3;17:3426. doi: 10.1038/s41467-026-70200-0 (PMC13076797; doi:10.1038/s41467-026-70200-0)
Supplement: Supplementary file 1 — Supplementary Information [file 41467_2026_70200_MOESM1_ESM.pdf]

Supplementary Information for

Climate's influence on topography encoded in stream network topology and geometry

Minhui Li[1, 2, 3, 4, †], Hansjörg Seybold[5, 6, †], Xudong Fu[1, 3, *], Baosheng Wu[1, 3],
Peter A. Raymond[4] and James W. Kirchner[2, 7, 8, *]

[1] Department of Hydraulic Engineering, Tsinghua University, Beijing, China

[2] Department of Environmental Systems Science, ETH Zurich, Zurich, Switzerland

[3] Key Laboratory of Hydrosphere Sciences of the Ministry of Water Resources, Tsinghua University, Beijing, China

[4] Yale School of the Environment, Yale University, New Haven, USA

[5] Department of Civil, Environmental and Geomatic Engineering, ETH Zurich, Zurich, Switzerland

[6] Institute for Interdisciplinary Mountain Research, Austrian Academy of Science, Innsbruck, Austria

[7] Swiss Federal Research Institute WSL, Birmensdorf, Switzerland

[8] Department of Earth and Planetary Science, University of California, Berkeley, CA, USA

[*]Corresponding authors: Xudong Fu (xdfu@tsinghua.edu.cn) and James Kirchner (kirchner@ethz.ch)

[†]These authors contributed equally: Minhui Li, Hansjörg Seybold.

Contents:

Texts S1-S3
Figures S1-S5
Tables S1-S4

Text S1. Topological metrics of stream networks

As an alternative to Tokunaga's parameter *c,* stream network topology may also be quantified by network-averaged differences in the Horton-Strahler order ($\overline{\Delta \text{HS}}$) between pairs of upstream tributaries. Tokunaga parameter *c* and $\overline{\Delta \text{HS}}$ are strongly correlated (Spearman ρ=0.63) among 11,946 5th-order Tokunaga self-similar stream networks analyzed in the main text. $\overline{\Delta \text{HS}}$ makes no self-similarity assumptions and provides a straightforward interpretation of stream network topology. Higher values of $\overline{\Delta \text{HS}}$ indicate more lower-order side-branches. $\overline{\Delta \text{HS}}$ is inherently scale-dependent and tends to increase with basin order (Fig. S1). In contrast, Tokunaga parameter *c* is normalized to account for scale effects (Fig. S1). Moreover, $\overline{\Delta \text{HS}}$ is strongly influenced by the large number of low-order streams, which dominate most stream networks. Consequently, $\overline{\Delta \text{HS}}$ may not fully capture the broader, scale-invariant properties of stream network topology. Nonetheless, bifurcation and side-branching angles exhibit broadly similar variation with $\overline{\Delta \text{HS}}$ (Fig. S4) as they do with Tokunaga parameter *c* (Fig. 4 in the main paper).

Text S2. Statistical test of Tokunaga self-similarity of stream networks

Testing Tokunaga self-similarity requires two steps[24], as Tokunaga scaling describes a specific exponential regularity in the side-branch structure of hierarchical networks, expressed through Tokunaga parameters (Equation 1 in the main text). Because Equation 1 presupposes a stable, scale-invariant hierarchy of stream orders, one must first test for ordinary self-similarity to ensure that branching structure is consistent across scales[23,24]. The side-branching ratio $T_{\omega,\omega+k}$ is independent of the order ω and depends only on the order difference, k[23,24](see also Methods in the main text), such that $T_{\omega,\omega+k} = T_k$. This condition provides a general definition of self-similarity in tree-like graphs[23,24,25]. Only once this global self-similarity is confirmed does it make sense to test for Tokunaga self-similarity, which concerns the finer-scale pattern of side-branching. Thus, some networks may be self-similar without exhibiting Tokunaga scaling (Fig. S2).

First, we test the self-similarity of a river network of order **Ω** using an analysis of variance (ANOVA) through the following **Ω**-2 Null-hypotheses[24]:

$\mathrm{H}_{0,1}$: $\mathrm{E}[n_{1,2}] = \mathrm{E}[n_{2,3}] =\ldots= \mathrm{E}[n_{(\Omega-2),(\Omega-1)}]= \mathrm{E}[n_{(\Omega-1),\Omega}]$

$\mathrm{H}_{0,2}$: $\mathrm{E}[n_{1,3}] = \mathrm{E}[n_{2,4}] =\ldots= \mathrm{E}[n_{(\Omega-2),\Omega}]$

...

$\mathrm{H}_{0,(\Omega-2)}$: $\mathrm{E}[n_{1,(\Omega-1)}]= \mathrm{E}[n_{2,\Omega}]$

Here $n_{i,j}$ (where $1 \le i < j \le \Omega$) consists of a series of $n_{i,j}^1, n_{i,j}^2, n_{i,j}^3, \cdots$, where each $n_{i,j}^l$ represents the number of streams of order i that join the nonterminal junctions of the l-th stream of order j. The case of streams joining the highest-order stream (with order **Ω**) includes a single value and hence their statistics, such as $n_{(\Omega-1),\Omega}$ and $n_{(\Omega-2),\Omega}$ are excluded. The statistical test is designed to achieve reasonable predictive power based on two assumptions: (1) the $n_{i,j}^l$ values corresponding to different triplets (i,j,l) are independent, and (2) for a fixed pair (i,j), the values $n_{i,j}^l$ can be regarded as a

sample from a random variable, denoted by $n_{i,j}$ (ref. *24*).

The second step tests Tokunaga self-similarity using the coefficient of determination (R^2) derived from the regression in Equation (4) in the main text. A river network is considered Tokunaga self-similar if the coefficient of determination exceeds 0.8 (ref. *24*).

About 86% of 16,322 5th-order stream networks from the NHDPlus-HR dataset passed the ANOVA self-similarity test at a significance level of p=0.05 (Fig. S2). Similar results are obtained using a non-parametric Kruskal-Wallis test instead of ANOVA (90% pass at p=0.05), confirming that our results are robust to the choice of statistical test. Networks that passed self-similarity (ANOVA test) were then tested for Tokunaga self-similarity using the coefficient of determination (R^2) of Tokunaga's scaling relation (Equation 4 in the main text). With an R^2 threshold of 0.8, 85% of the self-similar networks also meet the Tokunaga scaling criterion, resulting in 73% (11,946) of all 5th-order networks across the contiguous United States being Tokunaga self-similar (Fig. S2).

Text S3. Scale effects

Choosing the optimal network size to analyze involves trade-offs. On the one hand, networks that are lower-order, and thus smaller, can better characterize local variations in network topology and regional climatic conditions. On the other hand, networks that are higher-order, and hence larger, yield more precise estimates of topological parameters. However, these larger networks also average out variations in network topology, climate, and geological structure.

To explore the impact of varying stream network scales on our results, we additionally performed our analysis on 3,454 6th-order river networks in the NHDPlus-HR dataset[40]. Similar to our analysis of the 5th-order networks, we used Horton-Strahler order values provided by the NHDPlus-HR dataset and have excluded networks containing (i) more than 10% artificial flowlines (i.e., canals and pipelines), (2) channel slope uniformly equal to 0.00001 due to the dataset's slope cutoff. Approximately 76% of the 6th-order networks passed the ANOVA test, supporting the self-similarity hypothesis (compared to 86% of 5th-order networks; Fig. S2). The 2,635 networks that passed the ANOVA test were further evaluated for Tokunaga self-similarity scaling, using the coefficient of determination (R^2) with a threshold of 0.8 (ref. *24*). With this criterion, 2,417 (or approximately 70%) of all 6th-order networks also met both of the Tokunaga self-similarity criteria (compared to 73% of 5th-order networks; Fig. S2).

Across the 2,417 6th-order Tokunaga self-similar river networks, climatic aridity is uncorrelated with Tokunaga parameter c (ρ_{partial}=-0.01), but correlated with average channel slope (ρ_{partial}=0.33, p<0.0001), slope ratio (ρ_{partial}=-0.31, p<0.0001), and mean side-branching angle (ρ_{partial}=0.25, p<0.0001). These variables, in turn, have strong partial correlations with Tokunaga parameter c, suggesting that climate indirectly influences network topology through its effects on topography and network geometry (Table S1).

In the 6th-order networks, Tokunaga parameter c is significantly correlated with

network-averaged slope ratios ($\rho_{partial}$=-0.21, p<0.0001) and mean channel slope ($\rho_{partial}$=0.27, p<0.0001). While side-branching angles are strongly correlated with slope ratios ($\rho_{partial}$=-0.49, p<0.0001), their correlation with mean channel slope is much weaker ($\rho_{partial}$=-0.04). Notably, there is a substantial direct correlation between climatic aridity and side-branching angles ($\rho_{partial}$=0.25, p<0.0001), when topographic effects are factored out.

These results are consistent with the analyses of 5th-order networks presented in the main text, further confirming the robustness of our main findings.

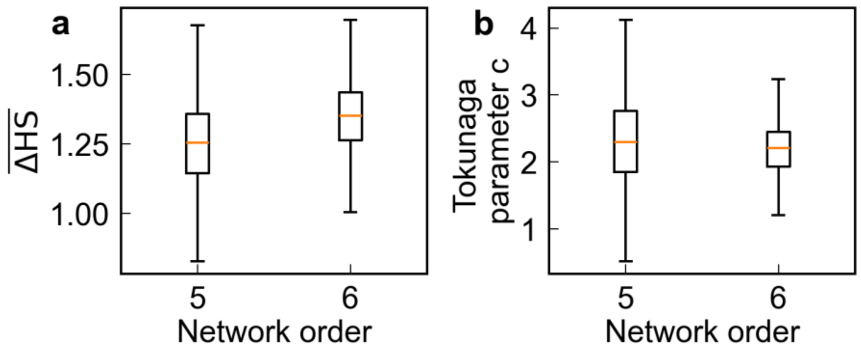

Fig. S1. Boxplots of (a) network-averaged Horton-Strahler order differences ($\overline{\mathbf{\Delta HS}}$) and (b) Tokunaga parameter *c* for 11,946 5th-order and 2,417 6th-order Tokunaga self-similar networks. Boxes indicate the interquartile range (IQR) and the red line denotes the median. Whiskers extend to 1.5 times the IQR; values beyond this range are considered outliers and are not shown.

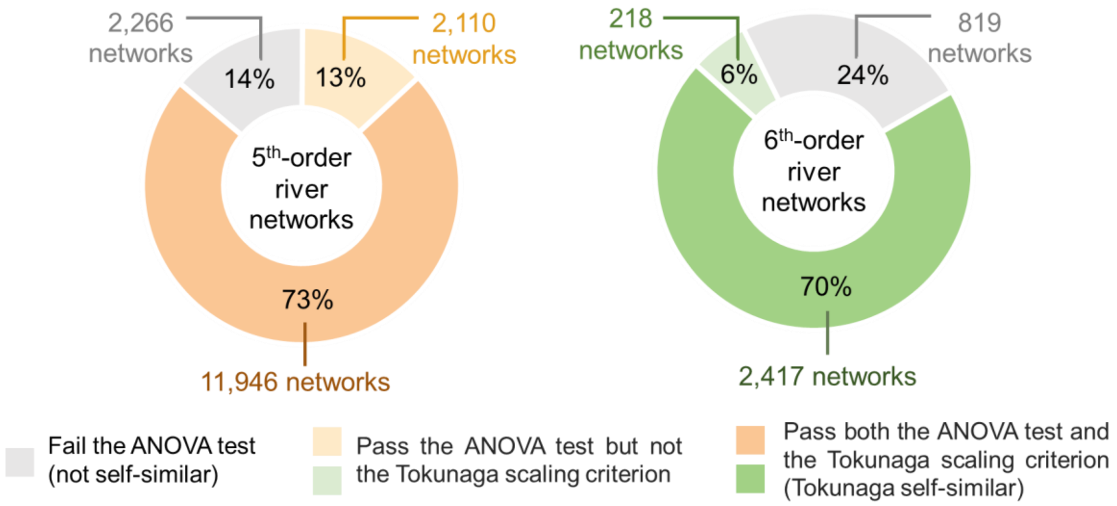

Fig. S2. Percentage of 5th- and 6th-order stream networks in different classes based on the self-similarity test. Among all 5th-order networks, 73% are Tokunaga self-similar (denoted in dark yellow; passing both the ANOVA test and the Tokunaga scaling criterion, following the method outlined in ref. *24*). 13% of all 5th-order networks pass the ANOVA test, but not the Tokunaga scaling criterion (light yellow), and 14% fail the ANOVA test and are thus not self-similar (grey color in the left pie chart). Among all 6th-order river networks, 70% are Tokunaga self-similar (dark green). 6% of all 6th-order networks pass the ANOVA test, but not the Tokunaga scaling criterion (light green), and 24% fail the ANOVA test and thus are not self-similar (grey color in the right pie chart).

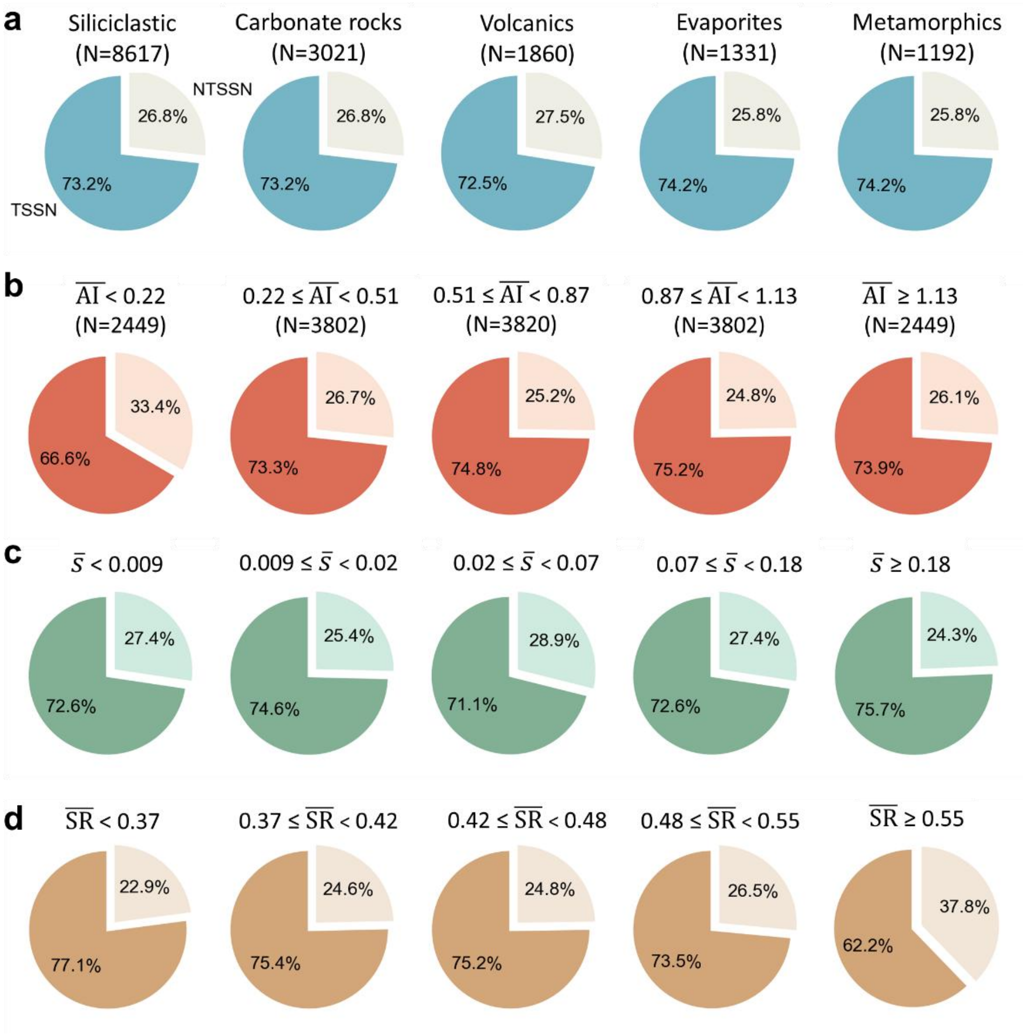

Fig. S3. Distributions of 5th-order stream network self-similarity types across different classes of (a) lithology, (b) network-averaged aridity index ($\overline{\mathrm{AI}}$), (c) network-averaged channel slope ($\bar{S}$), and (d) network-averaged slope ratio ($\overline{\mathrm{SR}}$). Stream networks are categorized into two types: Tokunaga self-similar networks (TSSN; dark colors) and non-Tokunaga self-similar networks (NTSSN; light colors). In panel (a), 301 networks with mixed or missing lithology data or classified as water body (see Methods in the main text) had to be excluded; these networks are included in panels (b-d). The number of networks in each lithology class is shown in brackets. In panels (b–d), networks are grouped into five classes: the lowest and highest classes represent values below the 15th percentile and above the 85th percentile, respectively. The remaining three classes contain approximately equal numbers of networks. The number of networks in each class is shown in brackets in panel (b). The fraction of TSSNs does not show systematic variation with (a) lithology or (c) channel slope. However, TSSNs are less frequent under arid conditions ($\overline{\mathrm{AI}}$<0.22) than in all other aridity categories ($\overline{\mathrm{AI}}$≥0.22; panel b). A slight decline in TSSN occurrence is observed as the slope ratio increases (d).

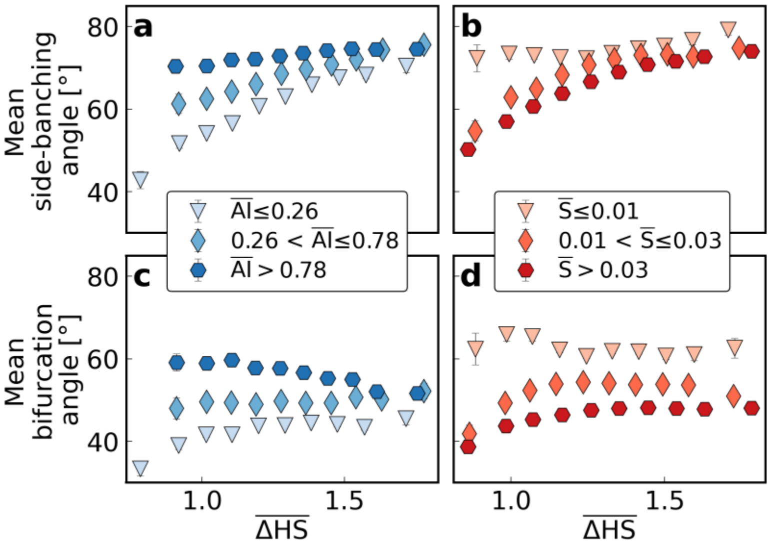

Fig. S4. Variation in mean side-branching angles (a-b) and bifurcation angles (c-d) with network-averaged differences in the Horton-Strahler order ($\overline{\Delta HS}$), across different classes of network-averaged aridity index ($\overline{AI}$) and channel slope ($\bar{S}$). The data are averaged within 10 bins. The first and last bins comprise $\overline{\Delta HS}$ values smaller than the 1st percentile and larger than the 99th percentile, respectively. The remaining eight bins are equally spaced between these percentiles. The error bars indicate the standard error of the mean for each bin, where these are larger than the plotting symbols. For both aridity index (a, c) and channel slope (b, d), the first class includes data with values smaller than the 20th percentile, the second class includes the 20th-50th percentiles, and the last class includes values above the 50th percentile.

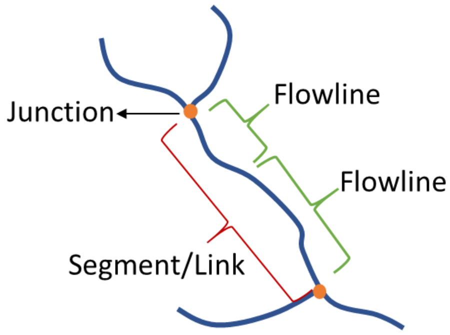

Fig. S5. Definitions of flowlines, river segments, and links in the high-resolution National Hydrographic Dataset. A river segment or link is a river section connecting two adjacent junctions or a channel head with its first downstream junction. Flowlines refer to individual components of river segments as defined by the National Hydrographic Dataset Plus High Resolution (NHDPlus-HR)[40].

Table S1. Spearman rank correlations (plain font, upper yellow triangle) and partial rank correlations (bold font, lower green triangle) for 2,417 6th-order Tokunaga self-similar networks, with (**) meaning p<0.01 and (***) meaning p<0.001. Partial rank correlations quantify the association between each pair of variables after removing any linear confounding effects from the other variables. Compared with Fig. 6 in the main text, which shows the same partial correlations for 11,946 5th-order Tokunaga self-similar stream networks, the overall correlation structure is consistent: aridity index (AI) is much more strongly correlated with mean slope ratio, mean channel slope, and mean side-branching angle, than with Tokunaga parameter *c*. Parameter *c* is correlated with mean slope ratio, mean channel slope, and mean side-branching angle. Mean slope ratios have a stronger influence on side-branching angles than mean channel slopes do.

	Tokunaga parameter c	Mean side-branching angle	Mean AI	Mean slope ratio	Mean channel slope
Tokunaga parameter c		0.24***	0.21***	-0.24***	0.14***
Mean side-branching angle	**0.09*****		0.48***	-0.69***	-0.28***
Mean AI	**-0.01**	**0.25*****		-0.45***	0.06**
Mean slope ratio	**-0.21*****	**-0.49*****	**-0.31*****		0.48***
Mean channel slope	**0.27*****	**-0.04****	**0.33*****	**0.50*****	

Table S2. Spearman rank correlations (plain font, upper yellow triangle) and partial rank correlations (bold font, lower green triangle) for 11,946 5th-order Tokunaga self-similar networks with less than 10% artificial lines, with p<0.001 for all cases. Partial rank correlations quantify the association between each pair of variables after removing any linear confounding effects from the other variables. Unlike Fig. 6 in the main text, Table S2 uses area ratio instead of slope ratio to quantify landscape dissection. Consistent with the main results shown in Fig. 6 of the main text, aridity index (AI) is much more strongly correlated with mean area ratio, mean channel slope, and mean side-branching angle, than with Tokunaga parameter *c*. Parameter *c* is correlated with mean area ratio, mean channel slope, and mean side-branching angle.

	Tokunaga parameter c	Mean side-branching angle	Mean AI	Mean area ratio	Mean channel slope
Tokunaga parameter c		0.25	0.12	-0.37	0.06
Mean side-branching angle	**0.20**		0.44	-0.28	-0.26
Mean AI	**-0.06**	**0.42**		-0.26	0.04
Mean area ratio	**-0.33**	**-0.10**	**-0.17**		0.06
Mean channel slope	**0.14**	**-0.31**	**0.18**	**0.06**	

Table S3. Spearman rank correlations (plain font, upper yellow triangle) and partial rank correlations (bold font, lower green triangle) for 11,515 5th-order Tokunaga self-similar networks with less than 5% artificial lines, with $p<0.001$. Partial rank correlations quantify the association between each pair of variables after removing any linear confounding effects from the other variables. Compared with Fig. 6 in the main text, which shows the partial correlations for networks with less than 10% (instead of 5%) artificial lines, the overall correlation structure is consistent: aridity index (AI) is much more strongly correlated with mean slope ratio, mean channel slope, and mean side-branching angle, than with Tokunaga parameter *c*. Parameter *c* is correlated with mean slope ratio, mean channel slope, and mean side-branching angle. Mean slope ratios have a stronger influence on side-branching angles than mean channel slopes do.

	Tokunaga parameter c	Mean side-branching angle	Mean AI	Mean slope ratio	Mean channel slope
Tokunaga parameter c		0.26	0.12	-0.25	0.05
Mean side-branching angle	**0.13**		0.44	-0.66	-0.24
Mean AI	**-0.06**	**0.23**		-0.44	0.06
Mean slope ratio	**-0.18**	**-0.49**	**-0.31**		0.40
Mean channel slope	**0.18**	**-0.05**	**0.29**	**0.41**	

Table S4. Spearman rank correlations (plain font, upper yellow triangle) and partial rank correlations (bold font, lower green triangle) for 12,378 5th-order Tokunaga self-similar networks with less than 20% artificial lines, with (*) meaning $p<0.05$ and (***) meaning $p<0.001$. Partial rank correlations quantify the association between each pair of variables after removing any linear confounding effects from the other variables. Compared with Fig. 6 in the main text, which shows the partial correlations for stream networks with less than 10% (rather than 20%) artificial lines, the overall correlation structure is consistent: aridity index (AI) is much more strongly correlated with mean slope ratio, mean channel slope, and mean side-branching angle, than with Tokunaga parameter *c*. Parameter *c* is correlated with mean slope ratio, mean channel slope, and mean side-branching angle. Mean slope ratios have a stronger influence on side-branching angles than mean channel slopes do.

	Tokunaga parameter c	Mean side-branching angle	Mean AI	Mean slope ratio	Mean channel slope
Tokunaga parameter c		0.23***	0.12***	-0.24***	0.07***
Mean side-branching angle	**0.12*****		0.43***	-0.64***	-0.29***
Mean AI	**-0.06*****	**0.23*****		-0.44***	0.02*
Mean slope ratio	**-0.19*****	**-0.45*****	**-0.32*****		0.43***
Mean channel slope	**0.20*****	**-0.10*****	**0.27*****	**0.41*****	
